# Use of Machine Learning and Artificial Intelligence Methods in Geriatric Mental Health Research Involving Electronic Health Record or Administrative Claims Data: A Systematic Review

**DOI:** 10.3389/fpsyt.2021.738466

**Published:** 2021-09-20

**Authors:** Mohammad Chowdhury, Eddie Gasca Cervantes, Wai-Yip Chan, Dallas P. Seitz

**Affiliations:** ^1^Department of Psychiatry, Cumming School of Medicine, University of Calgary, Calgary, AB, Canada; ^2^Department of Electrical and Computer Engineering, Queen's University, Kingston, ON, Canada

**Keywords:** geriatric, mental health, artificial intelligence, machine learning, administrative health data, electronic health records

## Abstract

**Introduction:** Electronic health records (EHR) and administrative healthcare data (AHD) are frequently used in geriatric mental health research to answer various health research questions. However, there is an increasing amount and complexity of data available that may lend itself to alternative analytic approaches using machine learning (ML) or artificial intelligence (AI) methods. We performed a systematic review of the current application of ML or AI approaches to the analysis of EHR and AHD in geriatric mental health.

**Methods:** We searched MEDLINE, Embase, and PsycINFO to identify potential studies. We included all articles that used ML or AI methods on topics related to geriatric mental health utilizing EHR or AHD data. We assessed study quality either by Prediction model Risk OF Bias ASsessment Tool (PROBAST) or Quality Assessment of Diagnostic Accuracy Studies (QUADAS-2) checklist.

**Results:** We initially identified 391 articles through an electronic database and reference search, and 21 articles met inclusion criteria. Among the selected studies, EHR was the most used data type, and the datasets were mainly structured. A variety of ML and AI methods were used, with prediction or classification being the main application of ML or AI with the random forest as the most common ML technique. Dementia was the most common mental health condition observed. The relative advantages of ML or AI techniques compared to biostatistical methods were generally not assessed. Only in three studies, low risk of bias (ROB) was observed according to all the PROBAST domains but in none according to QUADAS-2 domains. The quality of study reporting could be further improved.

**Conclusion:** There are currently relatively few studies using ML and AI in geriatric mental health research using EHR and AHD methods, although this field is expanding. Aside from dementia, there are few studies of other geriatric mental health conditions. The lack of consistent information in the selected studies precludes precise comparisons between them. Improving the quality of reporting of ML and AI work in the future would help improve research in the field. Other courses of improvement include using common data models to collect/organize data, and common datasets for ML model validation.

## Introduction

Geriatric mental health conditions such as depression or dementia are common, and it can be challenging to identify individuals with these conditions and predict outcomes associated with these conditions. Real-world data sources such as electronic health records (EHRs) and administrative health data (AHD) are increasingly used in geriatric mental health research. These data sources are available in many countries and health regions, and the details and information contained in these databases vary across the jurisdiction. The data contained in EHRs and administrative datasets are typically collected for the provision of medical care and purposing, such as financial reimbursement of providers. While this data is not collected primarily for health research purposes, EHRs and administrative data are frequently used for observational and epidemiological studies (sometimes referred to as outcomes studies). Given the non-randomized nature of studies utilizing EHR and AHD methods are used to minimize the risk of confounding and bias during the design and analysis of studies.

The typical analytic strategies employed with EHR and AHD studies involve multivariate regression models such as linear regression, logistic regression, or time-to-event models such as Cox-proportional hazards models. There is increasing interest in the potential applications of machine learning (ML) and artificial intelligence (AI) methods in mental health research (1) and in analyzing EHR and AHD data (2). ML and AI methods may provide benefits over standard biostatistical regression analysis when there is a high complexity to the underlying data (high dimensionality), which is becoming more common with EHR and AHD data sources as a greater range of information is included in these data sources (e.g., free-text clinical notes from EHR) and greater numbers of AHD sources are available for data linkage and analysis (e.g., laboratory, imaging, genetics) (3).

The application of ML and AI to the analysis of EHR and AHD in fields outside of geriatric mental health is increasing, including the development of recommendations for using ML and AI methods with these datasets (4), as well as studies of ML and AI in biomedical research (5). A recent review of EHR studies using ML or AI approaches for diagnosis or classification identified 99 unique publications across all clinical conditions (6). A review focused on the application of ML and AI approaches to dementia research using EHR identified five studies, although the review included data sources that were not routinely available in EHR and AHD, including neuroimaging or biomarker data (7). A review of the application of ML and AI approaches to research in mental health disorders, including all age groups, identified 28 studies, 6 of which utilized EHR data sources (1). To date, there are no reviews examining the application of ML and AI methods for studies using EHR and AHD in geriatric mental health research.

Our systematic review identifies the current application of ML and AI to EHR and AHD research in geriatric mental health. We identified the number of studies currently available in this field, the characteristics of study populations, data sources, and types of ML and AI methods used, along with potential strengths and limitations of studies, including the quality of study reporting. This review will highlight the current applications of ML and AI in geriatric mental health research and identify opportunities for future application of these methods to understanding geriatric mental health problems using these common data sources.

## Materials and Methods

### Research Question

To avoid the likelihood of missing relevant articles, the inquiry is recommended to be broad (8). For this review, our research question was: what research has been undertaken to apply machine learning and artificial intelligence methods to geriatric mental health conditions using EHR or AHD? We further sought to understand the types of geriatric mental conditions included in studies, the purpose of ML or AI approaches, information on sources of data used in studies, and assessments of study quality as part of our review.

### Data Sources and Searches

We conducted this review following a pre-specified protocol and in accordance with the Preferred Reporting Items for Systematic Reviews and Meta-analyses (PRISMA) reporting guidelines [(9); Supplementary Table 1]. We performed an extensive search using appropriate keywords and medical subject headings to find relevant studies. A predetermined search strategy was employed in relevant databases after consultation with a librarian. We searched MEDLINE, Embase, and PsycINFO (each from inception to April 2021) to identify studies with the assistance of a research librarian. Additionally, we explored the reference lists of all relevant studies for potentially relevant citations. The search strategy was based on five key domains: artificial intelligence, geriatric, mental health, electronic health records, and administrative health data. We used free-text words and Medical Subject Headings (MeSH) terms to identify all relevant studies for each key domain. Certain text words were truncated, or wildcards were used when required. The Boolean operators “AND” and “OR” were used to combine the words and MeSH terms so that the search yielded specific yet comprehensive results. The line-by-line search strategy employed in MEDLINE (Ovid) is provided in Supplementary Table 2.

### Eligibility Criteria

We set specific inclusion and exclusion criteria to eliminate irrelevant studies. Only original studies that focused on ML or AI in geriatric mental health using EHR or AHD data were included in this review. We excluded reviews, editorials, commentaries, protocols, conference abstracts, and letters to the editor. We considered all types of study designs, anticipating that ML or AI techniques may use different types of study design. There were also no restrictions on the languages of the studies. However, studies conducted in populations other than older adults were excluded, and we defined older adults as study populations where the average age of participants was 65 years and older.

Studies that did not use EHR or AMD data were also excluded. We considered EHR as studies involving health records from outpatient or inpatient settings where the data was directly extracted from clinical records. Data in EHR studies could include structured data such as diagnoses, medications, or procedures, as well as unstructured data such as free-text clinical notes. Information from studies reporting imaging results within EHR was included provided that the ML or AI methods were applied to information related to the ordering of imaging tests or interpretation of results (e.g., whether an imaging test was performed, or text contained in radiology reports) as this information is commonly available in EHR. We excluded studies that utilized ML or AI approaches to raw imaging data or genetic information, which is not routinely available in abstracted EHR data. Administrative health data included information related to diagnoses, clinical services, prescribed medication, hospitalizations, and emergency department visits. AHD does not typically include free-text clinical notes. Typically, multiple sources of AHD are linked across separate databases for studies using AHD in contrast to EHR data, where all data sources are available from a single EHR record. Studies including both EHR and AHD together were also included. Further, studies that did not consider a mental health issue were also excluded. We included major neurocognitive disorders and dementia in our definition of geriatric mental health conditions in addition to other mental health conditions such as depression, schizophrenia, and suicide. The complete list of terms used in our search strategy is provided in Supplementary Table 3.

### Study Selection

Four reviewers (MC, DS, EG, and GC) participated in the study selection and data extraction process. Eligible articles were identified independently by the reviewers using a two-step process. First, all articles identified from the search of electronic databases were exported to Covidence (10) to remove duplicate publications. Next, the title and abstracts of non-duplicated records were independently screened by two reviewers (MC and DS). All studies identified by one of the reviewers as potentially relevant were retained (based on eligibility criteria) and included in the full-text screening. Full-text articles were further screened for eligibility by the same two reviewers (MC and DS) independently. Lastly, the selected articles in the full-text review underwent data extraction, with each article reviewed by two of the four members of the review team. Any disagreement between reviewers was resolved through consensus.

### Data Extraction and Synthesis

For each selected article, two out of the four reviewers independently extracted data using Covidence (10). The following information from each study was extracted: study ID, country, the purpose of the ML/AI, study design, type of dataset used and data format, sample size, ML or AI methods used, predictors (features) used by ML/AI, comparison with other methods, the performance of ML/AI, and main finding(s). As we anticipated substantial heterogeneity in study designs, study populations, and methods, we did not plan to conduct a quantitative meta-analysis of results.

### Study Quality Assessment

ML or AI techniques are generally used for either prediction or diagnostic/classification purposes. Considering this, we assessed each study either by Prediction model Risk OF Bias ASsessment Tool [PROBAST; (11, 12)] or Quality Assessment of Diagnostic Accuracy Studies [QUADAS-2; (13)] checklist depending on the purpose of the selected study. Each reviewer independently assessed study quality.

PROBAST is designed to evaluate the risk of bias and concerns regarding the applicability of diagnostic and prognostic prediction model studies. PROBAST contains 20 questions under four domains: participants, predictors, outcome, and analysis, facilitating judgment of risk of bias and applicability. The overall risk of bias (ROB) of the prediction models was judged as “low”, “high”, or “unclear”, and overall applicability of the prediction models was considered as “low concern”, “high concern”, and “unclear” according to the PROBAST checklist (11, 12). When a prediction model evaluation is judged as low on all domains, then the model is treated as having “low ROB” or “low concern regarding applicability”. On the other hand, when model evaluation is judged as high for at least one domain, then the model is treated as having “high ROB” or “high concern regarding applicability”. Finally, when a prediction model evaluation is judged as unclear in one or more domains and is judged as low in the rest, then the model is treated as having “unclear ROB” or “unclear concern regarding applicability”.

QUADAS-2 (13) is the modified version of QUADAS, a tool used in systematic reviews to evaluate the risk of bias and applicability of diagnostic accuracy studies. QUADAS-2 consists of 11 signaling questions in four key domains: patient selection, index test(s), reference standard, and flow and timing. Signaling questions helps to judge the ROB as “low”, “high”, or “unclear”. Similar to PROBAST, when a study is judged as “low” on all domains, then the overall ROB and applicability of that study is judged as “low risk of bias” and “low concern regarding applicability”. However, if a study is rated “high” or “unclear” in one or more domains, the study may be classified as “at risk of bias” or “concerns regarding applicability”. Both PROBAST and QUADAS-2 are used to assess the risk of bias and concerns regarding applicability. In our review, we have used the tools for the assessment of the risk of bias.

### Data Analysis

Descriptive synthesis was undertaken to describe the existing literature on artificial intelligence techniques in geriatric mental health using AHD or EHR. Using the PRISMA flow diagram (14), we summarized the number of studies identified and those excluded (with the reason for exclusion) and included in the systematic review. The results of included studies were summarized in tables and synthesized in a narrative format.

## Results

### Study Identification and Selection

**Figure 1:**
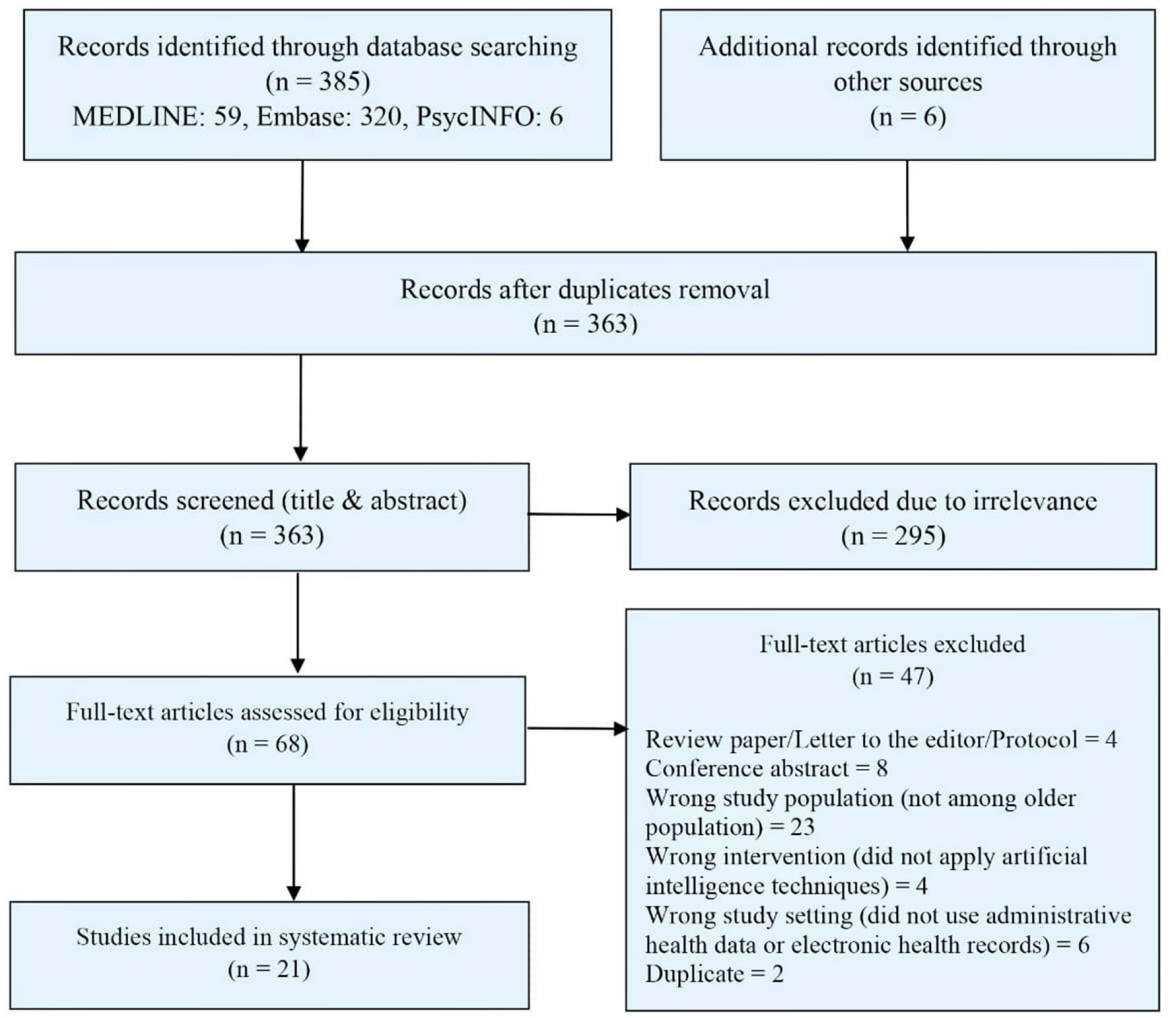
PRISMA diagram for the systematic review of studies on artificial intelligence in geriatric mental health using EHR or AHD.

We identified 385 articles through our electronic database search and an additional six articles through reference search. After removing duplicates, 363 titles and abstracts were screened for eligibility, and from there, 68 articles were selected for full-text screening. After assessing full-texts, 21 articles were finally selected for the systematic review (7, 15–34). The detailed study selection process is summarized in Figure 1.

### Study Characteristics

The detailed characteristics of the studies included in this review are presented in Table 1. Among the studies we identified, most of the studies were conducted in the USA (*N* = 12). The remaining studies were conducted in Spain (*N* = 3), UK (*N* = 2), South Korea (*N* = 2), France (*N* = 1), and Austria (*N* = 1). Study designs mainly were cohort (*N* = 14) followed by case-control studies (*N* = 5) and cross-sectional studies (*N* = 1). The study design was not reported in one study. The sample size of the individual studies varied between 1,909 and 17,227,820.

**Table 1:** Study characteristics of the included studies.

**Study ID**	**Country**	**Purpose of the ML/AI**	**Study design**	**Type of datasets used and data format**	**Sample size**	**ML or AI methods used**	**Predictors (features) used by ML/AI**	**Comparison with other methods**	**Performance of ML/AI**	**Main finding**
Kim et al. (15)	South Korea	Prediction of dementia	Cohort	AHD, structured	11,443	SVM, WEKA	Demographics, diagnoses, clinical measurements, laboratory values	Two sets of features compared	Longitudinal Model 1: Best accuracy = 72.0%	SVM approaches can be used to predict who will develop dementia
Nori et al. (16)	USA	Predict dementia incidence	Nested case-control	AHD, structured	Cases = 44,945, Controls = 760,646	Lasso, logistic regression	50 predictors	No	AUC = 0.693	On large datasets, ML methods can automatically recruit many predictors
Fisher et al. (27)	USA	Model progression of Alzheimer's disease	Cohort study	EHR, structured	1,909	Conditional Restricted Boltzmann Machine	44 variables, cognition laboratory, clinical information	RF	Accuracy, correlation coefficient, R square, AUC, and the root mean square errors	Predict disease progression of Alzheimer's disease
Wang et al. (29)	USA	Predict death among people with dementia	Cohort	EHR, structured and unstructured	26,921	DNN, LSTM, and NLP	500 features, patient demographic variables	No	AUC = 0.956 [0.955–0.956] (1-year model)	DNN can be accurate in predicting mortality and could be used as a proxy for selecting patients
Mar et al. (30)	Spain	Predict dementia-related neuropsychiatric symptoms	Cohort	EHR, unstructured	4,003	RF	62 variables including demographics, chronic disease, prescriptions	No	AUC = 0.80 for the psychotic cluster model, AUC = 0.74 for the depressive cluster model	Predict the presence of psychotic and/or depressive symptoms in dementia-diagnosed patients
Park et al. (31)	South Korea	Predict incidence of Alzheimer's disease	Cohort	AHD, structured	40,736	RF, SVM logistic regression	Demographics, lab tests, medication prescriptions, diagnoses	Logistic regression model	Best AUC of 0.898, 0.775, and 0.725 in predicting baseline, 1- and 4-year incident AD	RF outperformed SVM and logistic regression for every prediction year in predicting AD
Jauk et al. (32)	Austria	Identify patients at high risk for delirium	Cohort	EHR, structured	4,663	RF	Demographics, diagnoses, laboratory, nursing notes	Clinical expert risk scores	Sensitivity = 74.1%, Specificity = 82.2%, AUC = 0.86, Calibration = Poor	Demonstrated ML's predictive power for delirium
Miled et al. (7)	USA	Predict onset of dementia	Case-control	EHR, structured and unstructured	Training: 7,644, Testing: 17,760	RF	235 features, prescriptions, diagnosis, medical notes, demographics	No	The best model (highest accuracy) accuracy of 77.43%	Model which is generalizable and can predict dementia within 1 year
Nori et al. (33)	USA	Predict the risk for incident dementia and mild cognitive impairment	Nested case-control	AHD and EHR, structured	561,093 cases 16,666,727 controls	Gradient boosted trees, feed-forward network, recurrent neural network	Medications, diagnoses, procedures, demographics, and health service utilization	Comparison of deep learning and ML model	AUC on test data at year 0: 92.4% (BT), 93.1% (FFN), 94.4% (RNN); AUC on validation data at year 8: 79.9% (BT), 80.7% (FFN), 77.0% (RNN)	FFN performed best among the three deep network models, but marginally better than boosted trees
Mar et al. (34)	Spain	Identify clusters of depressive and psychotic symptoms of dementia	Cohort	AHD, structured	631,949	RF	Demographics, medical conditions, and medications	No	AUC = 0.8	Estimations of psychotic symptoms, depressive symptoms, incidence, prevalence of dementia
Tsang et al. (17)	UK	Predict hospitalization among dementia patients	Cohort	EHR, structured	59,298	Entropy regularization with ensemble DNN	54,649 unique features or event codes	RF	Accuracy = 0.759 (reduced features), Accuracy = 0.755 (full feature)	Method for predicting hospitalization of patients suffering from dementia
Anzaldi et al. (18)	USA	Determine the presence of geriatric syndromes and frailty	Cohort	AHD and EHR, structured and unstructured	18,341	NLP	Seed phrases for each syndrome	No	Correlations between frailty and NLP definitions of syndromes were between 0.07 and 0.27	Patients identified as “frail” had higher healthcare utilization and geriatric syndromes
Wang et al. (19)	USA	Identify important themes in provider's notes	Cohort	EHR, unstructured	7,875	Latent Dirichlet allocation topic model	250 topics	No	The trends of the topics from the clinical notes were compared to the structured data using Pearson's correlation	Analyze the disease course during the last 2 years of life
Halladay et al. (20)	USA	Develop a prediction rule for delirium	Cohort	EHR, structured	39,377	RF	16 features, demographics, cognition, infection, lab tests, diagnosis	Electronic delirium prediction rules	AUC compared to clinical rules = 0.81–0.91	A prevalent delirium prediction rule was developed
Kharrazi et al. (21)	USA	Assess the value of EHR to identify geriatric syndromes	Cohort	EHR, structured and unstructured	18,341	NLP	Unstructured electronic health record data (free-text clinical notes)	No	Sensitivity: 87.5–100% across the geriatric syndromes, Specificity: 95.4–100% across the geriatric syndromes	NLP method was incorporated in identification of individuals with geriatric syndromes
Chen et al. (22)	USA	Identify older adults automatically with geriatric syndromes from free text EHRs	Cohort	EHR, unstructured	18,341	End-to-end neural architecture, DNN	Diagnoses, target sentence, surrounding sentences, document	No	At sentence level: best model achieved a micro-F1 of 0.605. At patient level: best model achieved a micro-F1 of 0.843	EHR free text can be used to identify older adults with geriatric syndromes using proposed deep learning system
Violán et al. (23)	Spain	Identify multimorbidity pattern	Cross-section	EHR, structured	916,619	Fuzzy c-means cluster analysis	62–49, for a “no” to 2% prevalence threshold, respectively	No	O/E ratio and exclusivity was reported for different diseases within eight different patterns	Multimorbidity patterns were obtained in an elderly population
Shao et al. (24)	USA	Detection of dementia	Case-control	EHR, structured and unstructured	11,166	Topic modeling and logistic regression model	Total 853 features: non-dementia diagnoses, medications, and clinical notes	No	Optimal sensitivity = 0.825 and specificity = 0.832. AUC “Unclear = Dementia” = 0.912	Demonstrated automated methods feasibility to identify topics from EHR that can be used to assign a dementia risk score
Topaz et al. (25)	USA	Identify common neuropsychiatric symptoms of dementia	Cohort	EHR, structured and unstructured	89,459	NLP	Home healthcare free-text clinical notes	No	Average performance: F-score = 0.88, Precision = 0.87, Recall = 0.91	Identification of neuropsychiatric symptoms of dementia
Cabeli et al. (26)	France	Develop an approach to uncover relationships between mixed-type data from medical records	Not reported	EHR, structured	1,628	MIIC network learning algorithm	107 variables of different types (19 continuous and 88 categorical variables)	No	Not reported	Provides a user-friendly global visualization tool which could help other practitioners visualize and analyze effects from patient medical records
Ford et al. (28)	UK	Automatically detect undiagnosed dementia	Case-control	EHR, structured and unstructured	93,426	Logistic regression, naïve Bayes, RF	Eight diagnostic codes and nine keywords	Logistic regression	AUC Random Forest = 0.94, AUC Naive Bayes = 0.90, AUC Logistic Regression = 0.94 using both codes and keywords	Identified undiagnosed patients with dementia

*AHD, administrative health data; DNN, deep neural network; HER, electronic health record; ML, machine learning; NLP, natural language processing; RF, random forest; SVM, support vector machine; AUC, area under the curve; AD, Alzheimer's disease; LSTM, the long short-term memory; MIIC, multivariate information-based inductive causation*.

EHR was the most used data type and was used as the sole data source by 14 different studies. Five studies used only AHD, while two studies used both EHR and AHD. The dataset pattern was structured in nature in most studies (*N* = 11), unstructured free-text in only three studies, and seven studies used both structured and unstructured free-text data.

### ML and AI Methods Used in Studies

There were considerable variations among the specific ML or AI methods used by different studies. Random forest was the most common ML technique and was used by seven studies (7, 20, 28, 30–32, 34). Both natural language processing (NLP) (18, 21, 25, 29) and logistic regression modeling techniques (16, 24, 28, 31) were used by four studies. Three studies used deep learning approaches, including deep neural networks (17, 22, 29). Support vector machine (15, 31) and topic modeling (19, 24) were used by two studies each. Finally, lasso (33), naïve Bayes (28), multivariate information-based inductive causation (MIIC) network learning algorithm (26), fuzzy c-means cluster analysis (23), conditional restricted Boltzmann machine (27), WEKA (15), and gradient boosted trees (33) were applied by one study each.

Various sets of features, or sets of predictors, were considered in the identified studies. They include patient demographics, body measurements, history of family illness, personal disease history, administrative, diagnoses, laboratory, prescriptions, medications, medical notes, procedures, health service utilization, clinical and background information, topics in clinical notes, and ICD codes.

ML and AI methods were used for a variety of reasons. However, most of the studies applied the techniques either for prediction purposes (*N* = 9) or for classification or diagnosis purposes (*N* = 9). Among other objectives, one study used the methods to estimate the population incidence and prevalence of dementia and neuropsychiatric symptoms, one study to compare patients who are described in clinical notes as “frail” to other older adults concerning geriatric syndrome burden and healthcare utilization, and one study to compute and assess the significance of multivariate information between any combination of mixed-type variables and their relation to medical records.

Different outcomes were considered by different studies while incorporating ML or AI techniques. Major neurocognitive disorder, dementia, and Alzheimer's disease were the most common conditions among the articles included in our study and were reported in 11 studies (7, 15, 16, 24, 25, 27, 28, 30, 31, 33, 34). Among the other outcomes, a geriatric syndrome (falls, malnutrition, dementia, severe urinary control issues, absence of fecal control, visual impairment, walking difficulty, pressure ulcers, lack of social support, and weight loss) in 3 studies (18, 21, 22), delirium in 2 studies (20, 32), mild cognitive impairment (33), cognitive disorder (26), multimorbidity pattern (23), mortality (29), hospital admission (17), and themes/topics mentioned in care providers' notes (19) were considered once. Outcomes were primarily defined using ICD diagnosis codes in AHD and EHR.

The majority of the studies did not compare the ML and AI algorithms with any other biostatistical methods except a few where a comparison with logistic regression was made. ML/AI techniques outperformed logistic regression in one study while performed similarly in another study. The area under the receiver operating characteristics curve (AUC) was the most commonly reported performance measure of ML and AI algorithms, with values ranging from 0.69 to 0.98. None of the studies performed model validation in an external population where the performance of a model's prediction or classification can be generalized to unseen new data. As such, we could not evaluate any of the model's generalizability in this study.

### Study Quality Assessment

Study quality was assessed either by PROBAST or QUADAS-2, depending on the nature of the outcome. For example, in studies where the purpose was a prediction, we assessed quality with PROBAST. Nevertheless, when the study purpose was identification or classification, we assessed quality using QUADAS-2. Thus, PROBAST was applied in 11 studies, while QUADAS-2 was involved in 10 studies.

When PROBAST was applied, ROB was assessed as low in 100% of the studies according to the signaling questions of the “participants” domain, 91% of the studies according to the “predictors” domain, and 64% of the studies according to the “outcome” domain (Table 2). However, only in 36% of the studies ROB was assessed as low according to the “analysis” domain's signaling questions and was unclear in 55% of the studies. ROB was estimated as high in terms of both outcome and analysis in one study when PROBAST was applied. Only in three studies (16, 31, 34) low ROB was observed in all of the PROBAST domains.

**Table 2:** Study quality assessment using PROBAST.

**Study ID**	**Domain 1**.	**Domain 2**.	**Domain 3**.	**Domain 4**.
	**Participants**	**Predictors**	**Outcome**	**Analysis**
Kim et al. (15)	+	+	?	–
Nori et al. (16)	+	+	+	+
Fisher et al. (27)	+	+	?	?
Wang et al. (29)	+	+	+	?
Mar et al. (30)	+	+	+	?
Park et al. (31)	+	+	+	+
Jauk et al. (32)	+	+	?	?
Ben Miled et al. (7)	+	+	–	?
Nori et al. (33)	+	?	+	+
Mar et al. (34)	+	+	+	+
Tsang et al. (17)	+	+	+	?

*+ indicates low ROB; – indicates high ROB; ? indicates unclear ROB*.

When QUADAS-2 was applied, ROB was low in 80% of the studies according to the signaling questions of the “patient selection” domain (Table 3). However, in most of the studies, ROB was unclear according to the signaling questions of the other three domains of QUADAS-2. For example, ROB was unclear in 50% of the studies according to the “index test”, 60% of the studies according to the “reference standard”, and 80% of the studies according to the “flow and timing” domain's signaling questions. In each of the domains of QUADAS-2 except “flow and timing”, there was one study where ROB was assessed as high. In none of the studies, low ROB was observed according to all the QUADAS-2 domains.

**Table 3:** Study quality assessment using QUADAS-2.

**Study ID**	**Domain 1**.	**Domain 2**.	**Domain 3**.	**Domain 4**.
	**Patient selection**	**Index test(s)**	**Reference standard**	**Flow and timing**
Anzaldi et al. (18)	+	+	+	?
Wang et al. (19)	+	?	?	?
Halladay et al. (20)	+	–	+	+
Kharrazi et al. (21)	+	?	+	?
Chen et al. (22)	+	+	?	?
Violán et al. (23)	+	?	?	?
Shao et al. (24)	+	+	?	?
Topaz et al. (25)	+	+	–	+
Cabeli et al. (26)	?	?	?	?
Ford et al. (28)	–	?	?	?

*+ indicates low ROB; – indicates high ROB; ? indicates unclear ROB*.

## Discussion

Our review identified the application of ML and AI techniques in geriatric mental health using EHR or AHD data. We were able to identify 21 studies with all studies published recently within the past 5 years, suggesting the increasing application of ML and AI in this topic. As anticipated, ML or AI techniques were predominantly used either for prediction or classification purposes, and dementia was the most frequent condition considered in the studies. Both EHR and AHD data were considered; however, EHR data was the most frequent data source. There were considerable variations among ML, and AI techniques applied, ranging from more traditional ML techniques such as random forest to more advanced deep neural networks. The quality of study reporting was variable, with the majority of studies having unclear reporting of elements related to study quality. The relative advantages of ML or AI techniques compared to biostatistical methods were not assessed in most studies.

A recent review by Graham et al. (1) on a broader topic (AI for mental health and mental illness) identified 28 studies. However, their review is different from our systematic review in many different ways. First, the review by Graham et al. was not a systematic review, and the search was performed in PubMed and Google Scholar only with studies published between 2015 and 2019. In contrast, we performed a systematic review utilizing three databases without any time constraints. Second, their search was also not restricted to EHR and AHD data as ours; instead, they considered studies with data from all sources, including social media platforms, novel monitoring systems such as smartphone and video and brain imaging data. Third, their review included studies with participants from all the age groups starting from 14 years as opposed to our study, which focused on geriatric mental health where study participants were older adults. Fourth, neurocognitive disorders (e.g., dementia) were the primary outcome in most of our included studies. In contrast, Graham et al. did not consider studies with neurocognitive disorders in their review, and depression was identified as the most common mental illness. Nevertheless, supervised machine learning (e.g., random forest) was the most commonly used AI technique according to their review, similar to our findings. Another recent study by Elizabeta et al. (35) reviewed AI in the healthcare of older adults. They did not mention any specific number of studies; instead, they discussed some studies where ML or AI approaches were applied in the medical care of older people and concerns associated with AI use in medicine. However, the study is fundamentally different from our study in the sense that they consider overall healthcare, whereas our focus is only on mental health. Our review provides additional information about AI and ML in healthcare focused on the mental health of older adults and applications specific to EHR and AHD data sources.

EHR and AHD are rich resources that capture information of all the medical investigators involved in patients' healthcare records and provide ample opportunity to utilize this information for research, including mental health research. However, there are challenges associated with EHR and AHD data mainly due to the large sample sizes available, the volume of longitudinal data on participants, incompleteness, and inconsistency (6). Therefore, there is a potential role for automated analytic methods for disease diagnosis and prognosis or prediction from EHR and AHD data. Data-driven ML and AI techniques can overcome the challenges related to the volume, potential complexity, or dimensionality of EHR data. Information stored in EHR and AHD can be fall under two broad categories: structured (e.g., diagnosis, prescriptions, medical tests, etc.) and unstructured free texts [e.g., medical notes; (7)]. The use of structured data (i.e., diagnostic codes, prescription medications) is more extensive in many areas of health, primarily due to its limited pre-processing requirement compared to unstructured data. On the other hand, unstructured data primarily derived from medical notes poses additional challenges due to the difficulty of transferring free text into structured features. Nevertheless, unstructured data has also been applied to build models for different disease conditions, including dementia (7). NLP-based AI methods can translate unstructured text data into structured forms more amenable to machine inference or perform inference without explicit intervening translation. Combining structured and unstructured EHR data and using them to build ML models can produce better performance than each data source independently (7). Our study also noticed seven studies used combined data in predicting mortality and dementia and the diagnosis of geriatric syndromes and dementia.

Recently, increased emphasis has been put on using ML or AI tools in clinical research, particularly related to precision medicine (36, 37). Modern ML techniques offer benefits over traditional statistical methods due to their ability to detect complex non-linear relations, high-dimensional interactions among the features, and their capability to handle gigantic amounts of data. Since machine learning tools are more recent, advanced, and have the reputation of producing more accurate predictive performance, we anticipated studies using these tools might demonstrate improved predictive performance compared with the studies using more common biostatistical analytic methods. In our review, we identified only two studies (28, 31) comparing ML approaches with statistical methods. One study, Park et al. (31), found that the predictive performance of ML techniques random forest and support vector machine were superior compared to logistic regression in predicting Alzheimer's disease. In contrast, a similar predictive performance between random forest and naïve Bayes ML techniques and logistic regression was observed in predicting dementia in the study by Ford et al. (28). Overall this is in keeping with other findings that ML algorithms tend to provide mixed evidence for improving predictive performance compared with conventional statistical models in the other domains of health (38–42).

One of the considerations related to identifying situations where ML or AI may outperform biostatistical approaches include situations where the dataset is large and there are many complex and interrelated features or predictors. In situations where these conditions are not present, the performance of ML and AI techniques may not provide more accurate results when compared to biostatistical methods, even when they require additional expertise and computing resources to realize. While AI and ML may offer benefits in some situations, the potential limitations of these methods and optimal strategies for employing them in mental health research need to be carefully considered. Moreover, the inference of some ML models, such as neural networks, is hard to explain. Behavioral and performance explainability of ML models is a critical issue pertaining to whether “black box” models can be trusted, whether they appropriately infer from their input features, and whether they generalize well to “unseen” data (43).

Our review identified a lack of standard reporting in this area of literature. Authors often reported different aspects of the ML algorithms and in varying ways, which created difficulty for data collection and standardization within this review. In addition, the results of the ML study findings are often insufficiently reported primarily due to the inherent complexity of machine learning methods and the flexibility in specifying machine learning models, which hinders reliable assessment of study validity and consistent interpretation of study findings (5). Recently, new guidelines have been introduced with a list of reporting items to be included in a research article and a set of practical sequential steps to be followed for developing predictive models using ML (5). A new initiative to establish a TRIPOD (44) statement specific to machine learning (TRIPOD-ML) is also underway to guide authors to develop, evaluate and report ML algorithms properly (45). These reporting guidelines may assist authors in improving reporting in future studies in this area, particularly for research studies published in clinically oriented publications in contrast to engineering or computer science-focused publications.

The clinical implications of our findings include considerations related to the future application of ML and AI in geriatric mental health research (3, 46). ML and AI algorithms are typically used to classify or predict, translating to clinical applications related to diagnosis and prognosis (47). Mental health diagnoses are clinical compared to some other fields of medicine where diagnoses may be based on quantitative assessments or laboratory investigations. ML and AI analytic approaches may be well-suited to improve diagnostic accuracy in complex classification problems such as mental health diagnoses. To date, much of the research on this topic is confined to diagnosing dementia, although, as our review indicated, there is some research now related to the identification of geriatric syndromes or patterns of behavioral symptoms in dementia. ML and AI approaches require further study in diagnosing addictions and mental health problems in older adults. While only a few studies directly compare ML or AI approaches to more commonly used biostatistical methods, ML and AI may provide promising advances in disease state classification, particularly with more complex data.

Similarly, ML and AI may also provide improved prediction of the onset or progression of addictions and mental health problems. To date, the main clinical conditions that these methods have been applied to have been the onset of dementia. However, ML and AI approached may also facilitate improved prognostic models for predicting the onset of other geriatric mental health disorders. Predicting treatment response for an individual and personalizing therapeutic interventions based on this information, also known as precision medicine, is another potential application of ML and AI in geriatric mental health (47). Finally, ML and AI methods may be well-suited to analyzing unstructured data such as free-text clinical notes increasingly available in EHR. Incorporating clinician-generated data from unstructured data sources is likely to improve diagnostic or predictive performance when compared analyses conducted using only structured data such as diagnoses or laboratory values. Our review has identified current clinical applications of ML and AI approaches and highlights potential future areas for research and clinical applications related to research using EHR and AHD in geriatric mental health. While research on ML and AI in geriatric mental health is in its early stages it is anticipated that these methods will be increasingly used and have the potential to transform research and clinical care in this field as in other fields of medicine (48).

To the best of our knowledge, this is the first systematic review on the application of ML or AI in geriatric mental health conditions using EHR or AHD data, and a detailed critical appraisal of the applications has been performed. One of our study's strengths is the extent of the systematic search, which includes massive use of keywords and MeSH terms while searching three different databases and extensive use of the reference lists of the identified studies. We did not place any restrictions on language, geographical location, or time periods to keep our search broad. Consequently, there was little chance that any relevant study was missed. Nevertheless, our study also has limitations. We did not perform a search on gray literature. A search on electronic databases along with the gray literature could make the search more comprehensive. Although many of our identified studies were diagnostic or prognostic models and a meta-analysis of performance measures (e.g., AUC) of the models could provide a comprehensive summary of the performance of these models (49), we did not perform any meta-analysis from the studies due to their high heterogeneity. Failing to assess publication bias amongst the studies is another potential limitation of this study. Nevertheless, we assessed ROB associated with the studies using PROBAST and QUADAS-2 checklists.

In conclusion, we identified existing research on geriatric mental health in this study where ML or AI techniques were applied using EHR or AHD data. We were able to locate a relatively small number of studies that suggest ML or AI application in geriatric mental health is relatively uncommon at present, although this field is rapidly expanding throughout healthcare research. Outside of the clinical topic of dementia, there are few studies of other geriatric mental health conditions such as depression, anxiety, or suicide where ML and AI may be helpful. The lack of consistent information in the selected studies indicates that improvements in the quality of reporting of ML and AI in the future may also help improve research in this field. Additional information on how ML or AI approaches may be best utilized in EHR and AHD studies is required, including information about when these approaches are more or less likely to produce more accurate results compared to typical biostatistical analyses. Overall, ML and AI tools can play a vital role in redefining the diagnosis of mental illness using a secondary data source, thus facilitating early disease detection, a better understanding of disease progression, optimizing medication/treatment dosages, and uncovering novel treatments for geriatric mental health conditions.

## Author Contributions

All authors contributed to this work. DS contributed to the conception and design of the review. MC and DS read and screen abstracts and titles of potentially relevant studies, and screened the full-text papers. MC, DS, W-YC, and EC extracted data and rating the quality independently. MC performed the analysis and drafted the manuscript. DS and W-YC critically reviewed it and suggested amendments before submission. All authors approved the final version of the manuscript and take responsibility for the integrity of the reported findings.

## Funding

This research received no grant from any funding agency in public, commercial, or not-for-profit sectors. Partial support for this project was provided by the Canadian Institutes of Health Research—Canadian Consortium on Neurodegeneration in Aging.

## Conflict of Interest

The authors declare that the research was conducted in the absence of any commercial or financial relationships that could be construed as a potential conflict of interest.

## Publisher's Note

All claims expressed in this article are solely those of the authors and do not necessarily represent those of their affiliated organizations, or those of the publisher, the editors and the reviewers. Any product that may be evaluated in this article, or claim that may be made by its manufacturer, is not guaranteed or endorsed by the publisher.
