# Supplementary material for: Use of Machine Learning and Artificial Intelligence Methods in Geriatric Mental Health Research Involving Electronic Health Record or Administrative Claims Data: A Systematic Review
Source: Front Psychiatry. 2021 Sep 20;12:738466. doi: 10.3389/fpsyt.2021.738466 (PMC8488098; doi:10.3389/fpsyt.2021.738466)
Supplement: Supplementary file 1 [file Data_Sheet_1.docx]

**SUPPLEMENTARY MATERIAL**

**Table S1**

| **Section and Topic** | **Item #** | **Checklist item** | **Location where item is reported** |
| --- | --- | --- | --- |
| **TITLE** | | |  |
| Title | 1 | Identify the report as a systematic review. | Page 1 |
| **ABSTRACT** | | |  |
| Abstract | 2 | See the PRISMA 2020 for Abstracts checklist. | Page 2 |
| **INTRODUCTION** | | |  |
| Rationale | 3 | Describe the rationale for the review in the context of existing knowledge. | Page 3 |
| Objectives | 4 | Provide an explicit statement of the objective(s) or question(s) the review addresses. | Page 3 |
| **METHODS** | | |  |
| Eligibility criteria | 5 | Specify the inclusion and exclusion criteria for the review and how studies were grouped for the syntheses. | Page 4 & 5 |
| Information sources | 6 | Specify all databases, registers, websites, organisations, reference lists and other sources searched or consulted to identify studies. Specify the date when each source was last searched or consulted. | Page 4 & 5, |
| Search strategy | 7 | Present the full search strategies for all databases, registers and websites, including any filters and limits used. | Page 27-40, Supplementary Table S2 and S3 |
| Selection process | 8 | Specify the methods used to decide whether a study met the inclusion criteria of the review, including how many reviewers screened each record and each report retrieved, whether they worked independently, and if applicable, details of automation tools used in the process. | Page 5 & 6 |
| Data collection process | 9 | Specify the methods used to collect data from reports, including how many reviewers collected data from each report, whether they worked independently, any processes for obtaining or confirming data from study investigators, and if applicable, details of automation tools used in the process. | Page 5 & 6 |
| Data items | 10a | List and define all outcomes for which data were sought. Specify whether all results that were compatible with each outcome domain in each study were sought (e.g. for all measures, time points, analyses), and if not, the methods used to decide which results to collect. | Page 5 & 6 |
|  | 10b | List and define all other variables for which data were sought (e.g. participant and intervention characteristics, funding sources). Describe any assumptions made about any missing or unclear information. | Page 5 & 6 |
| Study risk of bias assessment | 11 | Specify the methods used to assess risk of bias in the included studies, including details of the tool(s) used, how many reviewers assessed each study and whether they worked independently, and if applicable, details of automation tools used in the process. | Page 5 & 6 |
| Effect measures | 12 | Specify for each outcome the effect measure(s) (e.g. risk ratio, mean difference) used in the synthesis or presentation of results. | N/A |
| Synthesis methods | 13a | Describe the processes used to decide which studies were eligible for each synthesis (e.g. tabulating the study intervention characteristics and comparing against the planned groups for each synthesis (item #5)). | N/A |
|  | 13b | Describe any methods required to prepare the data for presentation or synthesis, such as handling of missing summary statistics, or data conversions. | N/A |
|  | 13c | Describe any methods used to tabulate or visually display results of individual studies and syntheses. | Page 5 & 6 |
|  | 13d | Describe any methods used to synthesize results and provide a rationale for the choice(s). If meta-analysis was performed, describe the model(s), method(s) to identify the presence and extent of statistical heterogeneity, and software package(s) used. | N/A |
|  | 13e | Describe any methods used to explore possible causes of heterogeneity among study results (e.g. subgroup analysis, meta-regression). | N/A |
|  | 13f | Describe any sensitivity analyses conducted to assess robustness of the synthesized results. | N/A |
| Reporting bias assessment | 14 | Describe any methods used to assess risk of bias due to missing results in a synthesis (arising from reporting biases). | Page 5 & 6 |
| Certainty assessment | 15 | Describe any methods used to assess certainty (or confidence) in the body of evidence for an outcome. | N/A |
| **RESULTS** | | |  |
| Study selection | 16a | Describe the results of the search and selection process, from the number of records identified in the search to the number of studies included in the review, ideally using a flow diagram. | Page 6 & 7 |
|  | 16b | Cite studies that might appear to meet the inclusion criteria, but which were excluded, and explain why they were excluded. | Page 6 & 7 and Figure 1 |
| Study characteristics | 17 | Cite each included study and present its characteristics. | Page 6 & 7 and Table 1 |
| Risk of bias in studies | 18 | Present assessments of risk of bias for each included study. | Page 6 & 7 and Table 2 & Table 3 |
| Results of individual studies | 19 | For all outcomes, present, for each study: (a) summary statistics for each group (where appropriate) and (b) an effect estimate and its precision (e.g. confidence/credible interval), ideally using structured tables or plots. | Table 1 |
| Results of syntheses | 20a | For each synthesis, briefly summarise the characteristics and risk of bias among contributing studies. | Table 2 & Table 3 |
|  | 20b | Present results of all statistical syntheses conducted. If meta-analysis was done, present for each the summary estimate and its precision (e.g. confidence/credible interval) and measures of statistical heterogeneity. If comparing groups, describe the direction of the effect. | N/A |
|  | 20c | Present results of all investigations of possible causes of heterogeneity among study results. | N/A |
|  | 20d | Present results of all sensitivity analyses conducted to assess the robustness of the synthesized results. | N/A |
| Reporting biases | 21 | Present assessments of risk of bias due to missing results (arising from reporting biases) for each synthesis assessed. | Page 7 |
| Certainty of evidence | 22 | Present assessments of certainty (or confidence) in the body of evidence for each outcome assessed. | N/A |
| **DISCUSSION** | | |  |
| Discussion | 23a | Provide a general interpretation of the results in the context of other evidence. | Page 7-10 |
|  | 23b | Discuss any limitations of the evidence included in the review. | Page 9 & 10 |
|  | 23c | Discuss any limitations of the review processes used. | Page 9 & 10 |
|  | 23d | Discuss implications of the results for practice, policy, and future research. | Page 7-10 |
| **OTHER INFORMATION** | | |  |
| Registration and protocol | 24a | Provide registration information for the review, including register name and registration number, or state that the review was not registered. | N/A |
|  | 24b | Indicate where the review protocol can be accessed, or state that a protocol was not prepared. | N/A |
|  | 24c | Describe and explain any amendments to information provided at registration or in the protocol. | N/A |
| Support | 25 | Describe sources of financial or non-financial support for the review, and the role of the funders or sponsors in the review. | Page 11 |
| Competing interests | 26 | Declare any competing interests of review authors. | Page 11 |
| Availability of data, code and other materials | 27 | Report which of the following are publicly available and where they can be found: template data collection forms; data extracted from included studies; data used for all analyses; analytic code; any other materials used in the review. | N/A |

*From:*  Page MJ, McKenzie JE, Bossuyt PM, Boutron I, Hoffmann TC, Mulrow CD, et al. The PRISMA 2020 statement: an updated guideline for reporting systematic reviews. BMJ 2021;372:n71. doi: 10.1136/bmj.n71

For more information, visit: <http://www.prisma-statement.org/>

**Table S2.** Keywords used to search in MEDLINE

| **Domain/ Construct** | **#** | **Query** |
| --- | --- | --- |
| Artificial Intelligence | 1 | exp Artificial Intelligence/ |
|  | 2 | exp Machine Learning/ |
|  | 3 | artificial intelligence.tw,kf. |
|  | 4 | machine learning.tw,kf. |
|  | 5 | exp Supervised Machine Learning/ |
|  | 6 | exp Unsupervised Machine Learning/ |
|  | 7 | exp Deep Learning/ |
|  | 8 | exp Decision Trees/ |
|  | 9 | Decision tree*.tw,kf. |
|  | 10 | exp Neural Networks, Computer/ |
|  | 11 | Neural network*.tw,kf. |
|  | 12 | Deep neural network*.tw,kf. |
|  | 13 | Convolutional neural network*.tw,kf. |
|  | 14 | Random forest.tw,kf. |
|  | 15 | Reinforcement learning.tw,kf. |
|  | 16 | Gradient boosting.tw,kf. |
|  | 17 | exp Data Mining/ |
|  | 18 | exp Diagnosis, Computer-Assisted/ |
|  | 19 | Computer aided diagnosis.tw,kf. |
|  | 20 | Computational analysis.tw,kf. |
|  | 21 | exp Pattern Recognition, Automated/ |
|  | 22 | Multilayer perceptron*.tw,kf. |
|  | 23 | Bayes* network*.tw,kf. |
|  | 24 | exp Support Vector Machine/ |
|  | 25 | Support vector machine*.tw,kf. |
|  | 26 | k nearest neighbor*.tw,kf. |
|  | 27 | Naive bayes*.tw,kf. |
|  | 28 | Classification.mp. and regression tree*.tw,kf. [mp=title, abstract, original title, name of substance word, subject heading word, floating sub-heading word, keyword heading word, organism supplementary concept word, protocol supplementary concept word, rare disease supplementary concept word, unique identifier, synonyms] |
|  | 29 | Fuzzy.tw,kf. |
|  | 30 | Predictive modeling.tw,kf. |
|  | 31 | Outcome prediction.tw,kf. |
|  | 32 | exp Decision Support Systems, Clinical/ |
|  | 33 | Clinical decision support.tw,kf. |
|  | 34 | Computational intelligence.tw,kf. |
|  | 35 | exp Natural Language Processing/ |
|  | 36 | Natural language understanding.tw,kf. |
|  | 37 | Text mining.tw,kf. |
|  | 38 | 1 or 2 or 3 or 4 or 5 or 6 or 7 or 8 or 9 or 10 or 11 or 12 or 13 or 14 or 15 or 16 or 17 or 18 or 19 or 20 or 21 or 22 or 23 or 24 or 25 or 26 or 27 or 28 or 29 or 30 or 31 or 32 or 33 or 34 or 35 or 36 or 37 |
| Geriatric | 39 | Geriatric*.tw,kf. |
|  | 40 | exp Geriatric Psychiatry/ |
|  | 41 | Gerontology.tw,kf. |
|  | 42 | exp Geriatrics/ |
|  | 43 | Senior*.tw,kf. |
|  | 44 | exp Senior Centers/ |
|  | 45 | Elderly.tw,kf. |
|  | 46 | Old people*.tw,kf. |
|  | 47 | Old person*.tw,kf. |
|  | 48 | Old patient*.tw,kf. |
|  | 49 | Elder*.tw,kf. |
|  | 50 | exp “Aged, 80 and over”/ |
|  | 51 | exp Cognitive Aging/ |
|  | 52 | exp Frail Elderly/ |
|  | 53 | exp Homes for the Aged/ |
|  | 54 | exp Nursing Homes/ |
|  | 55 | Nursing home resident*.tw,kf. |
|  | 56 | Community dwelling older people*.tw,kf. |
|  | 57 | exp Health Services for the Aged/ |
|  | 58 | Aged care.tw,kf. |
|  | 59 | Aged care resident*.tw,kf. |
|  | 60 | exp Long-Term Care/ |
|  | 61 | Long-term care resident*.tw,kf. |
|  | 62 | Nursing home*.tw,kf. |
|  | 63 | Residential care.tw,kf. |
|  | 64 | 39 or 40 or 41 or 42 or 43 or 44 or 45 or 46 or 47 or 48 or 49 or 50 or 51 or 52 or 53 or 54 or 55 or 56 or 57 or 58 or 59 or 60 or 61 or 62 or 63 |
| Mental Health | 65 | exp Mental Health/ |
|  | 66 | exp Mental Disorders/ |
|  | 67 | Mental disorder*.tw,kf. |
|  | 68 | mental health.tw,kf. |
|  | 69 | exp Mental Competency/ |
|  | 70 | Mental competenc*.tw,kf. |
|  | 71 | Mental disability.tw,kf. |
|  | 72 | exp Intellectual Disability/ |
|  | 73 | Mental illness.tw,kf. |
|  | 74 | Mental disease.tw,kf. |
|  | 75 | Mental status.tw,kf. |
|  | 76 | Psychological disorder*.tw,kf. |
|  | 77 | Psychological well-being.tw,kf. |
|  | 78 | exp Depression/ |
|  | 79 | depression.tw,kf. |
|  | 80 | exp Anxiety/ |
|  | 81 | anxiety.tw,kf. |
|  | 82 | exp Stress, Psychological/ |
|  | 83 | Psychological effect*.tw,kf. |
|  | 84 | exp Persons with Mental Disabilities/ |
|  | 85 | exp Dementia/ or exp “Mental Status and Dementia Tests”/ |
|  | 86 | dementia.tw,kf. |
|  | 87 | exp Alzheimer Disease/ |
|  | 88 | Alzheimer.tw,kf. |
|  | 89 | exp Cognition Disorders/ |
|  | 90 | Cognition disorder*.tw,kf. |
|  | 91 | exp Cognitive Dysfunction/ |
|  | 92 | Cognitive dysfunction*.tw,kf. |
|  | 93 | 65 or 66 or 67 or 68 or 69 or 70 or 71 or 72 or 73 or 74 or 75 or 76 or 77 or 78 or 79 or 80 or 81 or 82 or 83 or 84 or 85 or 86 or 87 or 88 or 89 or 90 or 91 or 92 |
| Electronic Health Records | 94 | Electronic patient record*.tw,kf. |
|  | 95 | exp Electronic Health Records/ |
|  | 96 | Electronic medical record*.tw,kf. |
|  | 97 | Electronic health record*.tw,kf. |
|  | 98 | exp Medical Records Systems, Computerized/ |
|  | 99 | Computerized patient record*.tw,kf. |
|  | 100 | Computerized medical record*.tw,kf. |
|  | 101 | Computerized health record*.tw,kf. |
|  | 102 | Ambulatory medical record*.tw,kf. |
|  | 103 | Medical record*.tw,kf. |
|  | 104 | Health record*.tw,kf. |
|  | 105 | Hospital record*.tw,kf. |
|  | 106 | exp Health Records, Personal/ |
|  | 107 | Personal health record*.tw,kf. |
|  | 108 | Personal medical Record*.tw,kf. |
|  | 109 | Personal electronic health record*.tw,kf. |
|  | 110 | Electronic personal health record*.tw,kf. |
|  | 111 | Personal health information.tw,kf. |
|  | 112 | Medical Records System*.tw,kf. |
|  | 113 | Computerized Medical Records System*.tw,kf. |
|  | 114 | exp Patient Generated Health Data/ |
|  | 115 | 94 or 95 or 96 or 97 or 98 or 99 or 100 or 101 or 102 or 103 or 104 or 105 or 106 or 107 or 108 or 109 or 110 or 111 or 112 or 113 or 114 |
| Administrative Health Data | 116 | Administrative data.tw,kf. |
|  | 117 | Administrative claim data.tw,kf. |
|  | 118 | exp Insurance Claim Reporting/ |
|  | 119 | Administrative health data.tw,kf. |
|  | 120 | Electronic health data.tw,kf. |
|  | 121 | 116 or 117 or 118 or 119 or 120 |
|  | 122 | 115 or 121 |
|  | 123 | 38 and 64 and 93 and 122 |

**Table S3.**

**Keywords and search results from database MEDLINE.**

| **#** | **Query** | **Results from 19 April 2021** |
| --- | --- | --- |
| 1 | exp Artificial Intelligence/ | 110,593 |
| 2 | exp Machine Learning/ | 26,091 |
| 3 | artificial intelligence.tw,kf. | 13,051 |
| 4 | machine learning.tw,kf. | 42,269 |
| 5 | exp Supervised Machine Learning/ | 8,232 |
| 6 | exp Unsupervised Machine Learning/ | 456 |
| 7 | exp Deep Learning/ | 4,842 |
| 8 | exp Decision Trees/ | 11,471 |
| 9 | Decision tree*.tw,kf. | 10,032 |
| 10 | exp Neural Networks, Computer/ | 32,450 |
| 11 | Neural network*.tw,kf. | 59,355 |
| 12 | Deep neural network*.tw,kf. | 3,935 |
| 13 | Convolutional neural network*.tw,kf. | 10,125 |
| 14 | Random forest.tw,kf. | 9,264 |
| 15 | Reinforcement learning.tw,kf. | 3,801 |
| 16 | Gradient boosting.tw,kf. | 1,356 |
| 17 | exp Data Mining/ | 9,274 |
| 18 | exp Diagnosis, Computer-Assisted/ | 83,346 |
| 19 | Computer aided diagnosis.tw,kf. | 3,209 |
| 20 | Computational analysis.tw,kf. | 6,705 |
| 21 | exp Pattern Recognition, Automated/ | 25,735 |
| 22 | Multilayer perceptron*.tw,kf. | 1,772 |
| 23 | Bayes* network*.tw,kf. | 3,783 |
| 24 | exp Support Vector Machine/ | 7,443 |
| 25 | Support vector machine*.tw,kf. | 17,649 |
| 26 | k nearest neighbor*.tw,kf. | 3,013 |
| 27 | Naive bayes*.tw,kf. | 2,287 |
| 28 | Classification.mp. and regression tree*.tw,kf. [mp=title, abstract, original title, name of substance word, subject heading word, floating sub-heading word, keyword heading word, organism supplementary concept word, protocol supplementary concept word, rare disease supplementary concept word, unique identifier, synonyms] | 2,664 |
| 29 | Fuzzy.tw,kf. | 12,130 |
| 30 | Predictive modeling.tw,kf. | 2,216 |
| 31 | Outcome prediction.tw,kf. | 3,824 |
| 32 | exp Decision Support Systems, Clinical/ | 8,385 |
| 33 | Clinical decision support.tw,kf. | 5,672 |
| 34 | Computational intelligence.tw,kf. | 327 |
| 35 | exp Natural Language Processing/ | 4,604 |
| 36 | Natural language understanding.tw,kf. | 80 |
| 37 | Text mining.tw,kf. | 2,907 |
| 38 | 1 or 2 or 3 or 4 or 5 or 6 or 7 or 8 or 9 or 10 or 11 or 12 or 13 or 14 or 15 or 16 or 17 or 18 or 19 or 20 or 21 or 22 or 23 or 24 or 25 or 26 or 27 or 28 or 29 or 30 or 31 or 32 or 33 or 34 or 35 or 36 or 37 | 316,490 |
| 39 | Geriatric*.tw,kf. | 67,432 |
| 40 | exp Geriatric Psychiatry/ | 2,436 |
| 41 | Gerontology.tw,kf. | 4,247 |
| 42 | exp Geriatrics/ | 30,421 |
| 43 | Senior*.tw,kf. | 43,630 |
| 44 | exp Senior Centers/ | 205 |
| 45 | Elderly.tw,kf. | 260,234 |
| 46 | Old people*.tw,kf. | 4,231 |
| 47 | Old person*.tw,kf. | 1,164 |
| 48 | Old patient*.tw,kf. | 34,247 |
| 49 | Elder*.tw,kf. | 273,783 |
| 50 | exp "Aged, 80 and over"/ | 954,744 |
| 51 | exp Cognitive Aging/ | 783 |
| 52 | exp Frail Elderly/ | 12,289 |
| 53 | exp Homes for the Aged/ | 14,290 |
| 54 | exp Nursing Homes/ | 40,555 |
| 55 | Nursing home resident*.tw,kf. | 7,108 |
| 56 | Community dwelling older people*.tw,kf. | 1,206 |
| 57 | exp Health Services for the Aged/ | 17,967 |
| 58 | Aged care.tw,kf. | 3,104 |
| 59 | Aged care resident*.tw,kf. | 189 |
| 60 | exp Long-Term Care/ | 26,479 |
| 61 | Long-term care resident*.tw,kf. | 757 |
| 62 | Nursing home*.tw,kf. | 32,248 |
| 63 | Residential care.tw,kf. | 3,717 |
| 64 | 39 or 40 or 41 or 42 or 43 or 44 or 45 or 46 or 47 or 48 or 49 or 50 or 51 or 52 or 53 or 54 or 55 or 56 or 57 or 58 or 59 or 60 or 61 or 62 or 63 | 1,311,611 |
| 65 | exp Mental Health/ | 42,652 |
| 66 | exp Mental Disorders/ | 1,278,907 |
| 67 | Mental disorder*.tw,kf. | 50,104 |
| 68 | mental health.tw,kf. | 168,198 |
| 69 | exp Mental Competency/ | 8,411 |
| 70 | Mental competenc*.tw,kf. | 203 |
| 71 | Mental disability.tw,kf. | 675 |
| 72 | exp Intellectual Disability/ | 97,678 |
| 73 | Mental illness.tw,kf. | 30,836 |
| 74 | Mental disease.tw,kf. | 1,385 |
| 75 | Mental status.tw,kf. | 11,577 |
| 76 | Psychological disorder*.tw,kf. | 3,791 |
| 77 | Psychological well-being.tw,kf. | 10,054 |
| 78 | exp Depression/ | 126,046 |
| 79 | depression.tw,kf. | 359,909 |
| 80 | exp Anxiety/ | 90,523 |
| 81 | anxiety.tw,kf. | 208,930 |
| 82 | exp Stress, Psychological/ | 137,084 |
| 83 | Psychological effect*.tw,kf. | 4,382 |
| 84 | exp Persons with Mental Disabilities/ | 3,630 |
| 85 | exp Dementia/ or exp "Mental Status and Dementia Tests"/ | 177,334 |
| 86 | dementia.tw,kf. | 117,632 |
| 87 | exp Alzheimer Disease/ | 98,071 |
| 88 | Alzheimer.tw,kf. | 30,027 |
| 89 | exp Cognition Disorders/ | 98,753 |
| 90 | Cognition disorder*.tw,kf. | 405 |
| 91 | exp Cognitive Dysfunction/ | 21,407 |
| 92 | Cognitive dysfunction*.tw,kf. | 16,650 |
| 93 | 65 or 66 or 67 or 68 or 69 or 70 or 71 or 72 or 73 or 74 or 75 or 76 or 77 or 78 or 79 or 80 or 81 or 82 or 83 or 84 or 85 or 86 or 87 or 88 or 89 or 90 or 91 or 92 | 1,883,553 |
| 94 | Electronic patient record*.tw,kf. | 2,254 |
| 95 | exp Electronic Health Records/ | 22,277 |
| 96 | Electronic medical record*.tw,kf. | 19,045 |
| 97 | Electronic health record*.tw,kf. | 19,508 |
| 98 | exp Medical Records Systems, Computerized/ | 42,733 |
| 99 | Computerized patient record*.tw,kf. | 385 |
| 100 | Computerized medical record*.tw,kf. | 732 |
| 101 | Computerized health record*.tw,kf. | 22 |
| 102 | Ambulatory medical record*.tw,kf. | 53 |
| 103 | Medical record*.tw,kf. | 118,015 |
| 104 | Health record*.tw,kf. | 24,174 |
| 105 | Hospital record*.tw,kf. | 9,110 |
| 106 | exp Health Records, Personal/ | 2,117 |
| 107 | Personal health record*.tw,kf. | 1,172 |
| 108 | Personal medical Record*.tw,kf. | 82 |
| 109 | Personal electronic health record*.tw,kf. | 50 |
| 110 | Electronic personal health record*.tw,kf. | 124 |
| 111 | Personal health information.tw,kf. | 554 |
| 112 | Medical Records System*.tw,kf. | 444 |
| 113 | Computerized Medical Records System*.tw,kf. | 36 |
| 114 | exp Patient Generated Health Data/ | 94 |
| 115 | 94 or 95 or 96 or 97 or 98 or 99 or 100 or 101 or 102 or 103 or 104 or 105 or 106 or 107 or 108 or 109 or 110 or 111 or 112 or 113 or 114 | 176,137 |
| 116 | Administrative data.tw,kf. | 9,585 |
| 117 | Administrative claim data.tw,kf. | 17 |
| 118 | exp Insurance Claim Reporting/ | 4,589 |
| 119 | Administrative health data.tw,kf. | 518 |
| 120 | Electronic health data.tw,kf. | 276 |
| 121 | 116 or 117 or 118 or 119 or 120 | 14,761 |
| 122 | 115 or 121 | 189,431 |
| 123 | 38 and 64 and 93 and 122 | 58 |

**Keywords and search results from database Embase.**

| **#** | **Query** | **Results from 21 April 2021** |
| --- | --- | --- |
| 1 | exp artificial intelligence/ | 46,811 |
| 2 | exp machine learning/ | 245,181 |
| 3 | artificial intelligence.tw,kw. | 16,796 |
| 4 | machine learning.tw,kw. | 52,167 |
| 5 | exp supervised machine learning/ | 1,901 |
| 6 | exp unsupervised machine learning/ | 961 |
| 7 | exp deep learning/ | 14,684 |
| 8 | exp "decision tree"/ | 14,623 |
| 9 | Decision tree*.tw,kw. | 14,886 |
| 10 | exp artificial neural network/ | 53,609 |
| 11 | Neural network*.tw,kw. | 74,907 |
| 12 | Deep neural network*.tw,kw. | 4,616 |
| 13 | exp convolutional neural network/ | 8,538 |
| 14 | Convolutional neural network*.tw,kw. | 12,680 |
| 15 | exp random forest/ | 11,655 |
| 16 | Random forest.tw,kw. | 12,261 |
| 17 | Reinforcement learning.tw,kw. | 4,408 |
| 18 | Gradient boosting.tw,kw. | 1,717 |
| 19 | exp data mining/ | 14,412 |
| 20 | exp computer assisted diagnosis/ | 1,171,806 |
| 21 | Computer aided diagnosis.tw,kw. | 4,595 |
| 22 | Computational analysis.tw,kw. | 8,029 |
| 23 | exp automated pattern recognition/ | 16,789 |
| 24 | exp multilayer perceptron/ | 848 |
| 25 | exp Bayesian network/ | 1,264 |
| 26 | Bayes* network*.tw,kw. | 4,823 |
| 27 | exp support vector machine/ | 24,418 |
| 28 | Support vector machine*.tw,kw. | 22,290 |
| 29 | exp k nearest neighbor/ | 3,759 |
| 30 | k nearest neighbor*.tw,kw. | 3,779 |
| 31 | Naive bayes*.tw,kw. | 2,899 |
| 32 | Classification.mp. and regression tree*.tw,kw. [mp=title, abstract, heading word, drug trade name, original title, device manufacturer, drug manufacturer, device trade name, keyword, floating subheading word, candidate term word] | 3,605 |
| 33 | exp fuzzy c means clustering/ | 227 |
| 34 | Fuzzy.tw,kw. | 15,359 |
| 35 | Predictive modeling.tw,kw. | 2,802 |
| 36 | Outcome prediction.tw,kw. | 6,091 |
| 37 | exp clinical decision support system/ | 3,532 |
| 38 | Clinical decision support.tw,kw. | 7,487 |
| 39 | Computational intelligence.tw,kw. | 468 |
| 40 | exp natural language processing/ | 6,223 |
| 41 | Natural language understanding.tw,kw. | 91 |
| 42 | Text mining.tw,kw. | 3,247 |
| 43 | 1 or 2 or 3 or 4 or 5 or 6 or 7 or 8 or 9 or 10 or 11 or 12 or 13 or 14 or 15 or 16 or 17 or 18 or 19 or 20 or 21 or 22 or 23 or 24 or 25 or 26 or 27 or 28 or 29 or 30 or 31 or 32 or 33 or 34 or 35 or 36 or 37 or 38 or 39 or 40 or 41 or 42 | 1,503,085 |
| 44 | exp geriatric nursing/ or exp geriatric disorder/ or exp geriatric patient/ | 79,677 |
| 45 | Geriatric*.tw,kw. | 86,323 |
| 46 | exp gerontopsychiatry/ | 7,678 |
| 47 | Geriatric Psychiatry.tw,kw. | 2,914 |
| 48 | exp gerontology/ | 3,265 |
| 49 | Gerontology.tw,kw. | 5,502 |
| 50 | exp geriatrics/ | 38,611 |
| 51 | exp senior center/ | 401 |
| 52 | Senior*.tw,kw. | 61,814 |
| 53 | Elderly.tw,kw. | 376,348 |
| 54 | Old people*.tw,kw. | 6,126 |
| 55 | Old person*.tw,kw. | 1,732 |
| 56 | Old patient*.tw,kw. | 50,726 |
| 57 | Elder*.tw,kw. | 395,969 |
| 58 | Aged, 80.mp. and over.tw,kw. [mp=title, abstract, heading word, drug trade name, original title, device manufacturer, drug manufacturer, device trade name, keyword, floating subheading word, candidate term word] | 2,531 |
| 59 | exp cognitive aging/ | 1,926 |
| 60 | exp frail elderly/ | 10,543 |
| 61 | exp home for the aged/ | 11,087 |
| 62 | exp nursing home/ | 54,191 |
| 63 | Nursing home resident*.tw,kw. | 9,044 |
| 64 | Community dwelling older people*.tw,kw. | 1,453 |
| 65 | exp elderly care/ | 76,666 |
| 66 | Health Services for the Aged.tw,kw. | 432 |
| 67 | Aged care.tw,kw. | 3,617 |
| 68 | Aged care resident*.tw,kw. | 208 |
| 69 | exp long term care/ | 1,890,106 |
| 70 | Long-term care resident*.tw,kw. | 1,015 |
| 71 | Nursing home*.tw,kw. | 41,637 |
| 72 | Residential care.tw,kw. | 4,760 |
| 73 | 44 or 45 or 46 or 47 or 48 or 49 or 50 or 51 or 52 or 53 or 54 or 55 or 56 or 57 or 58 or 59 or 60 or 61 or 62 or 63 or 64 or 65 or 66 or 67 or 68 or 69 or 70 or 71 or 72 | 2,498,812 |
| 74 | exp mental health/ | 173,142 |
| 75 | exp mental disease/ | 2,295,240 |
| 76 | Mental disorder*.tw,kw. | 55,047 |
| 77 | mental health.tw,kw. | 209,101 |
| 78 | exp mental capacity/ | 83,958 |
| 79 | Mental competenc*.tw,kw. | 288 |
| 80 | exp mental deficiency/ | 137,054 |
| 81 | Mental disability.tw,kw. | 1,015 |
| 82 | exp intellectual impairment/ | 535,183 |
| 83 | exp mental disease/ | 2,295,240 |
| 84 | Mental illness.tw,kw. | 40,742 |
| 85 | Mental status.tw,kw. | 21,847 |
| 86 | Psychological disorder*.tw,kw. | 5,470 |
| 87 | Psychological well-being.tw,kw. | 12,768 |
| 88 | exp depression/ | 497,314 |
| 89 | depression.tw,kw. | 506,847 |
| 90 | exp anxiety/ | 227,309 |
| 91 | anxiety.tw,kw. | 303,085 |
| 92 | exp mental stress/ | 86,620 |
| 93 | Psychological effect*.tw,kw. | 5,534 |
| 94 | exp mentally disabled person/ | 1,256 |
| 95 | exp dementia/ | 381,155 |
| 96 | dementia.tw,kw. | 176,136 |
| 97 | exp Alzheimer disease/ | 212,542 |
| 98 | Alzheimer.tw,kw. | 42,873 |
| 99 | exp cognitive defect/ | 520,019 |
| 100 | Cognition disorder*.tw,kw. | 1,071 |
| 101 | Cognitive dysfunction*.tw,kw. | 25,624 |
| 102 | 74 or 75 or 76 or 77 or 78 or 79 or 80 or 81 or 82 or 83 or 84 or 85 or 86 or 87 or 88 or 89 or 90 or 91 or 92 or 93 or 94 or 95 or 96 or 97 or 98 or 99 or 100 or 101 | 2,810,236 |
| 103 | exp electronic patient record/ | 2,946 |
| 104 | Electronic patient record*.tw,kw. | 5,542 |
| 105 | exp electronic health record/ | 23,037 |
| 106 | Electronic health record*.tw,kw. | 28,635 |
| 107 | exp electronic medical record/ | 60,395 |
| 108 | Electronic medical record*.tw,kw. | 41,523 |
| 109 | exp electronic medical record system/ | 1,617 |
| 110 | Computerized patient record*.tw,kw. | 627 |
| 111 | Computerized medical record*.tw,kw. | 1,098 |
| 112 | Computerized health record*.tw,kw. | 28 |
| 113 | Ambulatory medical record*.tw,kw. | 65 |
| 114 | exp medical record/ | 269,486 |
| 115 | Health record*.tw,kw. | 35,056 |
| 116 | Hospital record*.tw,kw. | 13,993 |
| 117 | Personal health record*.tw,kw. | 1,410 |
| 118 | Personal medical Record*.tw,kw. | 111 |
| 119 | Personal electronic health record*.tw,kw. | 55 |
| 120 | Electronic personal health record*.tw,kw. | 158 |
| 121 | Personal health information.tw,kw. | 628 |
| 122 | Medical Records System*.tw,kw. | 918 |
| 123 | Computerized Medical Records System*.tw,kw. | 86 |
| 124 | Patient Generated Health Data.tw,kw. | 172 |
| 125 | 103 or 104 or 105 or 106 or 107 or 108 or 109 or 110 or 111 or 112 or 113 or 114 or 115 or 116 or 117 or 118 or 119 or 120 or 121 or 122 or 123 or 124 | 291,748 |
| 126 | Administrative data.tw,kw. | 13,293 |
| 127 | Administrative claim data.tw,kw. | 25 |
| 128 | Insurance Claim Reporting.tw,kw. | 17 |
| 129 | Administrative health data.tw,kw. | 729 |
| 130 | Electronic health data.tw,kw. | 353 |
| 131 | 126 or 127 or 128 or 129 or 130 | 14,211 |
| 132 | 125 or 131 | 304,111 |
| 133 | 43 and 73 and 102 and 132 | 300 |

**Keywords and search results from database PsycINFO.**

| **#** | **Query** | **Results from 26 April 2021** |
| --- | --- | --- |
| 1 | exp Artificial Intelligence/ | 22,964 |
| 2 | exp Machine Learning/ | 10,512 |
| 3 | artificial intelligence.tw. | 5,667 |
| 4 | machine learning.tw. | 7,730 |
| 5 | Supervised Machine Learning.tw. | 222 |
| 6 | exp Unsupervised Learning/ | 242 |
| 7 | Deep Learning.tw. | 1,743 |
| 8 | Decision tree*.tw. | 1,868 |
| 9 | Decision trees.mp. | 1,119 |
| 10 | exp Neural Networks/ | 33,465 |
| 11 | Neural network*.tw. | 19,701 |
| 12 | exp Deep Neural Networks/ | 466 |
| 13 | Deep neural network*.tw. | 539 |
| 14 | Convolutional neural network*.tw. | 711 |
| 15 | Random forest.tw. | 682 |
| 16 | exp Computational Reinforcement Learning/ | 260 |
| 17 | Gradient boosting.tw. | 92 |
| 18 | exp Data Mining/ | 3,639 |
| 19 | exp Computer Assisted Diagnosis/ | 1,592 |
| 20 | Computer aided diagnosis.tw. | 124 |
| 21 | exp Computational Modeling/ | 6,635 |
| 22 | Computational analysis.tw. | 392 |
| 23 | exp "Pattern Recognition (Computer Science)"/ | 1,540 |
| 24 | Multilayer perceptron*.tw. | 291 |
| 25 | exp Machine Learning Algorithms/ | 910 |
| 26 | Bayes* network*.tw. | 836 |
| 27 | Support Vector Machine.tw. | 1,948 |
| 28 | Support Vector Machine.mp. | 2,100 |
| 29 | k nearest neighbor*.tw. | 396 |
| 30 | Naive bayes*.tw. | 450 |
| 31 | Classification.mp. and regression tree*.tw. [mp=title, abstract, heading word, table of contents, key concepts, original title, tests & measures, mesh] | 423 |
| 32 | exp Fuzzy Logic/ | 1,644 |
| 33 | Fuzzy.tw. | 5,139 |
| 34 | Predictive modeling.tw. | 366 |
| 35 | Outcome prediction.tw. | 610 |
| 36 | exp Decision Support Systems/ | 3,406 |
| 37 | Clinical decision support.tw. | 472 |
| 38 | Computational intelligence.tw. | 287 |
| 39 | exp Natural Language Processing/ | 557 |
| 40 | Natural language understanding.tw. | 104 |
| 41 | Text mining.tw. | 683 |
| 42 | 1 or 2 or 3 or 4 or 5 or 6 or 7 or 8 or 9 or 10 or 11 or 12 or 13 or 14 or 15 or 16 or 17 or 18 or 19 or 20 or 21 or 22 or 23 or 24 or 25 or 26 or 27 or 28 or 29 or 30 or 31 or 32 or 33 or 34 or 35 or 36 or 37 or 38 or 39 or 40 or 41 | 80,168 |
| 43 | exp Geriatric Psychiatry/ | 1,582 |
| 44 | Geriatric*.tw. | 18,357 |
| 45 | exp Gerontology/ | 9,756 |
| 46 | Gerontology.tw. | 4,522 |
| 47 | exp Geriatrics/ | 14,128 |
| 48 | Geriatrics.tw. | 3,461 |
| 49 | Senior*.tw. | 29,338 |
| 50 | exp Elder Care/ | 4,965 |
| 51 | Senior Centers.tw. | 468 |
| 52 | exp Geriatric Patients/ | 13,661 |
| 53 | Elderly.tw. | 65,028 |
| 54 | exp Older Adulthood/ | 6,976 |
| 55 | Old people*.tw. | 1,734 |
| 56 | Old person*.tw. | 551 |
| 57 | Old patient*.tw. | 7,109 |
| 58 | Elder*.tw. | 74,147 |
| 59 | Aged, 80.mp. and over.tw. [mp=title, abstract, heading word, table of contents, key concepts, original title, tests & measures, mesh] | 229 |
| 60 | exp Cognitive Aging/ | 1,069 |
| 61 | Frail Elderly.mp. | 2,503 |
| 62 | Frail Elderly.tw. | 1,029 |
| 63 | Homes for the Aged.mp. | 2,815 |
| 64 | exp Nursing Homes/ | 9,137 |
| 65 | Nursing home resident*.tw. | 3,816 |
| 66 | exp Nursing Home Residents/ | 2,592 |
| 67 | Community dwelling older people*.tw. | 384 |
| 68 | Health Services for the Aged.mp. | 2,583 |
| 69 | Mental Health Services for the Aged.mp. | 38 |
| 70 | Aged care.tw. | 1,352 |
| 71 | Aged care resident*.tw. | 73 |
| 72 | exp Long Term Care/ | 5,428 |
| 73 | Long-term care resident*.tw. | 338 |
| 74 | Nursing home*.tw. | 12,589 |
| 75 | Residential care.tw. | 4,242 |
| 76 | 43 or 44 or 45 or 46 or 47 or 48 or 49 or 50 or 51 or 52 or 53 or 54 or 55 or 56 or 57 or 58 or 59 or 60 or 61 or 62 or 63 or 64 or 65 or 66 or 67 or 68 or 69 or 70 or 71 or 72 or 73 or 74 or 75 | 156,004 |
| 77 | exp Mental Health/ | 71,537 |
| 78 | exp Mental Disorders/ | 888,185 |
| 79 | Mental disorder*.tw. | 66,426 |
| 80 | mental health.tw. | 201,667 |
| 81 | Mental Competency.mp. | 2,270 |
| 82 | Mental competenc*.tw. | 220 |
| 83 | Mental disability.tw. | 616 |
| 84 | exp Intellectual Development Disorder/ | 45,633 |
| 85 | Intellectual Disability.mp. | 24,798 |
| 86 | Mental illness.tw. | 43,110 |
| 87 | Mental disease.tw. | 2,203 |
| 88 | Mental status.tw. | 4,814 |
| 89 | Psychological disorder*.tw. | 5,013 |
| 90 | Psychological well-being.tw. | 14,192 |
| 91 | exp "Depression (Emotion)"/ | 26,000 |
| 92 | depression.tw. | 265,783 |
| 93 | exp Anxiety/ | 77,096 |
| 94 | anxiety.tw. | 208,357 |
| 95 | exp Psychological Stress/ | 9,078 |
| 96 | Psychological effect*.tw. | 5,157 |
| 97 | Persons with Mental Disabilities.mp. | 99 |
| 98 | exp Dementia/ | 80,667 |
| 99 | dementia.tw. | 68,623 |
| 100 | exp Alzheimer's Disease/ | 48,669 |
| 101 | Alzheimer.tw. | 13,732 |
| 102 | exp Cognitive Impairment/ | 38,854 |
| 103 | Cognition Disorders.mp. | 31,841 |
| 104 | Cognitive Dysfunction.mp. | 9,871 |
| 105 | Cognitive dysfunction*.tw. | 7,658 |
| 106 | 77 or 78 or 79 or 80 or 81 or 82 or 83 or 84 or 85 or 86 or 87 or 88 or 89 or 90 or 91 or 92 or 93 or 94 or 95 or 96 or 97 or 98 or 99 or 100 or 101 or 102 or 103 or 104 or 105 | 1,274,431 |
| 107 | Electronic patient record*.tw. | 208 |
| 108 | exp Electronic Health Records/ | 894 |
| 109 | Electronic medical record*.tw. | 1,984 |
| 110 | Electronic health record*.tw. | 2,141 |
| 111 | Medical Records Systems, Computerized.mp. | 562 |
| 112 | Computerized patient record*.tw. | 24 |
| 113 | Computerized medical record*.tw. | 70 |
| 114 | Computerized health record*.tw. | 3 |
| 115 | Ambulatory medical record*.tw. | 0 |
| 116 | exp Medical Records/ | 4,256 |
| 117 | Medical record*.tw. | 10,484 |
| 118 | Health record*.tw. | 2,970 |
| 119 | Hospital record*.tw. | 1,063 |
| 120 | Health Records, Personal.mp. | 128 |
| 121 | Personal health record*.tw. | 220 |
| 122 | Personal medical Record*.tw. | 9 |
| 123 | Personal electronic health record*.tw. | 6 |
| 124 | Electronic personal health record*.tw. | 40 |
| 125 | exp Health Information/ | 2,502 |
| 126 | Personal health information.tw. | 148 |
| 127 | Medical Records System*.tw. | 42 |
| 128 | Computerized Medical Records System*.tw. | 1 |
| 129 | Patient Generated Health Data.mp. | 10 |
| 130 | 107 or 108 or 109 or 110 or 111 or 112 or 113 or 114 or 115 or 116 or 117 or 118 or 119 or 120 or 121 or 122 or 123 or 124 or 125 or 126 or 127 or 128 or 129 | 18,460 |
| 131 | Administrative data.tw. | 2,769 |
| 132 | Administrative claim data.tw. | 4 |
| 133 | Insurance Claim Reporting.mp. | 130 |
| 134 | Administrative health data.tw. | 71 |
| 135 | Electronic health data.tw. | 19 |
| 136 | 131 or 132 or 133 or 134 or 135 | 2,964 |
| 137 | 130 or 136 | 21,254 |
| 138 | 42 and 76 and 106 and 137 | 6 |
